# Clinical Characteristics and Risk Factors for Early versus Late Pulmonary Embolism in Trauma Patients: A Retrospective, Observational Study

**DOI:** 10.2147/IJGM.S387880

**Published:** 2022-10-21

**Authors:** Tariq Siddiqui, Mohammad Asim, Khalid Ahmed, Saji Mathradikkal, Zeenat Bakhsh, Maarij Masood, Ammar Al-Hassani, Syed Nabir, Nadeem Ahmed, Gustav Strandvik, Ayman El-Menyar, Hassan Al-Thani

**Affiliations:** 1 Trauma Surgery Section, Hamad General Hospital (HGH), Doha, Qatar; 2 Clinical Research, Trauma & Vascular Surgery, Hamad General Hospital, Doha, Qatar; 3 Radiology Department, Hamad General Hospital, Doha, Qatar; 4 Clinical Medicine, Weill Cornell Medical College, Doha, Qatar

**Keywords:** pulmonary embolism, trauma, timing, risk factors, outcomes

## Abstract

**Background:**

We sought to evaluate the clinical characteristics and risk factors for early versus late pulmonary embolism (PE) in trauma patients.

**Methods:**

This was a retrospective analysis of injured patients who presented with a confirmed PE between 2013 and 2019. Data were analysed and compared for patients with early PE (≤4 days) versus late PE (>4 days post-trauma).

**Results:**

The study included 82 consecutive trauma patients with confirmed diagnosis of PE. The mean age of patients was 42.3 ± 16.2 years. The majority were males (79.3%) and the median time from injury to PE was 10 days. Of the PE cases, 24 (29.3%) had early PE, while 58 (70.7%) had late PE. The early PE group had higher rates of surgical intervention within 24 hours of admission than the late PE group (p = 0.001). Also, the rate of sub-segmental thrombi was significantly higher in the early PE group (p = 0.01). The late PE group sustained more moderate-to-severe injuries ie, GCS ED <13 (p = 0.03) and the median time from injury to PE diagnosis was 15 days (p = 0.001). After adjusting for the potential covariates, surgery within 24 hours of admission [adjusted odds ratio 37.58 (95% confidence interval 3.393–416.20), p = 0.003] was found to be significant independent predictor of early PE in trauma patients.

**Conclusion:**

One-third of post-trauma PEs occurs early after trauma and the surgical intervention within the first 24 hours of admission is a major risk factor. A prospective study is needed to develop an objective risk assessment for the prevention and detection of early and late PE post-trauma.

## Introduction

Pulmonary embolism (PE) among trauma patients is relatively uncommon and is associated with serious complications and a higher rate of mortality.^1^,^2^ The reported incidence of PE ranges between 0.11% and 2.3% among patients who sustain traumatic injuries.^3^ The occurrence of deep vein thrombosis (DVT) and PE have been estimated to be 1.16% and 0.93%, respectively in patients with orthopedic trauma.^4^ There is emerging evidence that a transient hypercoagulable state exists due to the increased generation of thrombin and fibrin soon after trauma, which could develop the thromboembolism.^5^,^6^ It has been shown that the DVT and PE are two distinct entities as some patients with PE do not develop identifiable DVT or died prior to full investigation.^5–8^ However, in some cases the DVT may completely dislodge from its site of origin, leaving no residual DVT.

Around 10% of in-hospital deaths were related to pulmonary emboli, and autopsy studies revealed that two-thirds were missed.^9^ PE is the third most common cause of death in trauma patients who survived the initial 24 hours after injury.^10^,^11^ Ho et al reported fatal PE as the most common cause of late mortality.^12^ Severity of injuries, co-morbidity including body mass index (BMI) are important risk factors for fatal PE after major trauma.

Notably, early PE is known entity which is often diagnosed within 3 to 4 days post trauma.^13–15^ Previous studies have demonstrated that up to half of PEs occur within the first 4 days of hospitalization.^14^,^16^,^17^ It was found that early PE is associated with a significantly higher risk of early mortality as opposed to late PE.^18^ Of note, the occurrence of early PE can be predicted in trauma patients requiring ICU admission, particularly in older age, patients with long bone fractures and those with severe injury.^19^ Long bone extremity fractures were identified as the sole independent risk factor for early PE, whereas late PE groups had a higher injury severity score (ISS), severe head injury, severe chest injury, and a delay in chemical prophylaxis initiation beyond the first 24 hours.^16^ In recognition of these factors, there is increased focus on the preventability of early PE. Currently, data on the timing of PE among trauma patients and its clinical relevance are lacking in our trauma centre. Therefore, we sought to determine the clinical features, potential risk factors and outcomes of trauma patients in relation to the timing PE (early vs late PE). We believe that a better understanding of risk factors of PE will help risk-stratification and timely prevention and management.

## Methods

This was a retrospective chart review performed at the only level I trauma centre at Hamad General Hospital in Qatar. Data were retrieved from the Qatar national trauma registry, and our electronic patient record system (CERNER) for all injured patients who had a confirmed diagnosis of PE on computed tomography pulmonary angiography (CTPA) between July 2013 and April 2019. In our institution, the diagnosis of PE is confirmed after radiologist review of images from multidetector CT scanners with features of filing defects or obstruction in the pulmonary artery and its branches. Briefly, all patients were examined using Siemens SOMATOM Sensation (Siemens AG, Munich, Germany) 16‑ or 64‑slice machine. For all examinations, kV was set as 100, collimation of 0.6, rotation time of 0.5 s, slice thickness of 5 mm, and pitch of 1.0. The non-ionic intravenous contrast (100 mL of Omnipaque 350 mg/mL) was injected at a rate of 4.0–5.0 mL/s. Pulmonary arterial phase bolus tracking through the main pulmonary trunk was carried out, followed by aortic phase immediately following pulmonary arterial phase. Moreover, three‑dimensional reconstruction was performed, if indicated.^20^

The presence of an endoluminal central filling defect partially or totally occluding the pulmonary arteries was characterized radiologically as PE.^20^ Depending on the location of the emboli identified on the CT scan, the PE was classified as proximal or distal. Proximal PE referred to emboli found within the main or lobar arteries, while emboli that are segmental or sub-segmental were considered distal PE.^21^ Patients who had PE diagnosed within 4 days of hospitalization were classified as early PE. Late PE referred to cases diagnosed after 4 days of hospitalization, based on previous publications.^14^,^16^,^17^

Data included demographic characteristics, mechanism of injury, pre-existing comorbidities, anatomic location of injuries, injury severity score, abbreviated injury scores, Glasgow coma score (GCS) at the emergency department, clinical presentation, initial vitals, time from injury to PE, risk factors, echocardiography and CTPA findings, plasma D-dimer, cardiac Troponin, Shock Index, PE clinical scoring systems (Wells Score, Revised Geneva score, Simplified PESI), DVT on ultrasonography and on follow-up after PE, timing of venous thromboembolism (VTE) prophylaxis, treatment with anticoagulants, thrombolytic therapy, and thrombectomy. We also looked for the use of tranexamic acid (TXA) administration, blood transfusion, massive transfusion, ICU length of stay, ventilatory days, hospital length of stay (LOS), in-hospital complications, and mortality.

All trauma patients received chemical VTE prophylaxis except for those who were at a higher risk of bleeding. These included high grade solid organ injuries managed conservatively, head injuries with intracranial bleeding, spinal fractures with hematoma and spinal canal compromise, pelvic fracture with hematoma and venous bleeding, uncontrolled coagulopathy and massive transfusion (MT) requirement, chemoprophylaxis was delayed until corrected these risk factors were ameliorated. In patients with head injury, neurosurgery consultation was obtained prior to initiation of chemical VTE prophylaxis. Pneumatic compression devices were used in all patients on-admission until contraindicated. In our institution, the need for chemical prophylaxis is assessed by emergency physicians after team discussion. Notably, during the study period Doppler ultrasonography was not a routine practice for screening for DVT. However, post-PE Doppler studies were performed to rule-out DVT as the source of PE.

### Ethical Approval

The Institutional Review Board (MRC-01-19-269) of the Hamad Medical Corporation (Doha, Qatar) approved and granted exempt status for this retrospective study with a waiver of consent as there is no direct contact with patients and data were collected anonymously. The study complies with the Declaration of Helsinki.

### Statistical Analysis

Data were reported as a percentage, mean (± standard deviation), median, and range, where applicable. Student’s *t*-test was used to compare continuous variables, and a chi-square test was used to compare categorical variables for univariate analysis. PE cases were divided into 2 groups based on the time of PE diagnosis from the time of injury as early PE (≤4 days of injury) or late PE (>4 days). Demographic and injury-associated characteristics, type of prophylaxis for PE, complication, management, and outcomes were analysed according to early and late PE. Multivariate regression analysis was performed to identify independent predictors of early PE after adjusting for relevant covariates such as age, gender, BMI, surgery (within 24 hours), long bone fracture, GCS, ISS and massive transfusion (MT). Data were expressed using odds ratio and 95% confidence interval (CI). A 2-tailed p-value less than 0.05 was considered as statistically significant. The Statistical Package for the Social Sciences (SPSS) for Windows V.21.0 (SPSS, Chicago, Illinois, USA) was used for the data analysis.

## Results

The study cohort comprised of 82 consecutive patients with radiologically confirmed diagnosis of PE which constituted around 0.9% of the total trauma hospitalizations. The mean age of patients was 42.3 ± 16.2 years. The majority were males (79.3%). The median time from injury to PE was 10 days. Twenty-four (29.3%) were identified as early PE of which 3 cases were immediate PE diagnosed on initial CT scan, while 58 (70.7%) had a late PE. Blunt trauma was the most frequent injury mechanism as motor vehicle crash (MVC) (39%) followed by pedestrian hit (14.6%) and then falls from height (12.2%). Hypertension (14.6%), diabetes mellitus (13.4%), and hypercholesteremia (12.2%) were the most frequent pre-existing comorbidities. The most frequently injured anatomic regions were long bone fractures (67.1%), followed by chest (36.6%), abdomen (28.0%) and head (25.6%). Table 1 shows the comparison of demographics and clinical characteristics of trauma patients presenting with PE (early vs late). No significant difference was observed between the two groups for age, gender, BMI, mechanism of injury, pre-existing co-morbidities, associated injuries, injury severity score, and abbreviated injury scores. Compared with the early PE group, the late PE group was more likely to have hypercholesteremia (17.2% vs 0.0%; p = 0.03), sustained more moderate-to-severe injuries such as GCS ED <13 (37.0% vs 12.5%; p = 0.03); the median time from injury to PE diagnosis was 15 days.

**Table 1:** Demographics, and Injury Characteristics of Trauma Patients with Pulmonary Embolism (PE)

Variables	Overall (n=82)	Early PE (≤4 Days) n=24	Late PE (>4 Days) n=58	P value
**Age (mean±SD)**	42.3±16.2	40.9±14.8	42.9±16.8	0.60
**Males**	65 (79.3%)	19 (79.2%)	46 (79.3%)	0.98
**Body mass index (mean±SD)**	27.9±5.4	28.5±6.1	27.7±5.1	0.55
Time from injury to PE (days)*	10 (0–163)	3 (0–4)	15 (5–163)	0.001
**Mechanism of Injury:**				
Motor vehicle crash	32 (39.0%)	14 (58.3%)	18 (31.0%)	0.09 for all
Pedestrian injury	12 (14.6%)	1 (4.2%)	11 (19.0%)	
Bike/motorcycle	4 (4.9%)	2 (8.3%)	2 (3.4%)	
Fall from height	10 (12.2%)	3 (12.5%)	7 (12.1%)	
Struck by heavy Object	6 (7.3%)	2 (8.3%)	4 (6.9%)	
Unknown/others	18 (22.0%)	2 (8.3%)	16 (27.6%)	
**Co-morbidities:**				
Hypertension	12 (14.6%)	3 (12.5%)	9 (15.5%)	0.72
Diabetes	11 (13.4%)	2 (8.3%)	9 (15.5%)	0.38
Hypercholesteremia	10 (12.2%)	0 (0.0%)	10 (17.2%)	0.03
Coronary artery disease	2 (2.4%)	0 (0.0%)	2 (3.4%)	0.35
Smoking (n=77)	9 (11.7%)	3 (13.6%)	6 (10.9%)	0.73
**Associated injuries:**				
Long bone fractures	55 (67.1%)	17 (70.8%)	38 (65.5%)	0.58
Chest	30 (36.6%)	11 (45.8%)	19 (32.8%)	0.26
Abdomen	23 (28.0%)	9 (37.5%)	14 (24.1%)	0.46
Head	21 (25.6%)	3 (12.5%)	17 (29.3%)	0.10
Spine	24 (29.3%)	6 (25.0%)	18 (31.0%)	0.49
Pelvis	22 (26.8%)	8 (33.3%)	14 (24.1%)	0.48
**ISS**	18.8±12.5	18.7±11.7	18.8±12.9	0.98
**ISS ≥16**	41 (57.7%)	12 (50.0%)	29 (50.0%)	0.71
**GCS ED <13**	20 (28.6%)	3 (12.5%)	17 (37.0%)	0.03

**Abbreviations**: SD, standard deviation; ISS, injury severity score; GCS, Glasgow coma scale; ED, emergency department; *, median and range.

Table 2 shows the clinical presentations of and risk factors for trauma-associated PE. Most patients had symptomatic presentation indicating PE (77.8%), whereas 22.2% were asymptomatic. Dyspnea (54.9%), chest pain (45.1%) and unilateral lower limb pain (20.7%) were the most prominent symptoms in patients diagnosed with PE. The commonly observed risk factors included surgery (>24 hours; 45.1%), bed bound status (30.5%), surgery (<24 hours, 13.4%) and neck central line placement (20.7%). Also, deficiency of protein C (12.2%), protein S (12.2%), and antithrombin III (8.5%) were the most common thrombophilic disorders.

**Table 2:** Clinical Presentation and Risk Factors of Pulmonary Embolism (PE)

Variables	Overall (n=82)	Early PE (≤4 Days) n=24	Late PE (>4 Days) n=58	P value
**Clinical Presentation:**				
Asymptomatic	18 (22.2%)	9 (37.5%)	9 (15.8%)	0.03 for all
Symptomatic:	63 (77.8%)	15 (62.5%)	48 (84.2%)	
- Dyspnea	45 (54.9%)	9 (37.5%)	36 (62.1%)	0.04
- Chest pain	37 (45.1%)	7 (29.2%)	30 (51.7%)	0.06
- Unilateral lower limb pain	17 (20.7%)	9 (37.5%)	8 (13.8%)	0.01
- Syncope	6 (7.3%)	1 (4.2%)	5 (8.6%)	0.48
- Hemoptysis	5 (6.1%)	3 (12.5%)	2 (3.4%)	0.11
**Risk factors:**				
Surgery (>24 h)	37 (45.1%)	7(29.2%)	30 (51.7%)	0.06
Bed ridden	25 (30.5%)	6 (25.0%)	19 (32.8%)	0.48
Surgery (<24 h) *	11 (13.4%)	9 (37.5%) **	2 (3.4%)	0.001
Neck central line	17 (20.7%)	3 (12.5%)	14 (24.1%)	0.23
History of Warfarin	13 (15.9%)	0 (0.0%)	13 (22.4%)	0.01
Femoral central line	10 (12.2%)	2 (8.3%)	8 (13.8%)	0.49
History of DVT	8 (9.8%)	0 (0.0%)	8 (13.8%)	0.05
History of PE	1 (1.2%)	0 (0.0%)	1 (1.7%)	0.51
Paraplegia	8 (9.8%)	2 (8.3%)	6 (10.3%)	0.78
**Laboratory findings:**				
Protein C deficiency	10 (12.2%)	4 (16.7%)	6 (10.3%)	0.42
Protein S deficiency	10 (12.2%)	4 (16.7%)	6 (10.3%)	0.42
Anti-thrombin III deficiency	7 (8.5%)	1 (4.2%)	6 (10.3%)	0.36
Lupus anticoagulant	3 (3.7%)	0 (0.0%)	3 (5.2%)	0.25

**Notes**: *3 had immediate PE so only 21 early PE cases considered for post-surgery <24h; **8/9 were orthopedic surgery; DVT; deep vein thrombosis.

The early PE group had a higher rate of unilateral lower limb pain (37.5% vs 13.8%; p = 0.01) and were more likely to undergo surgery within 24 hours of admission (37.5% vs 3.4%; p = 0.001). Patients with late PE were more likely to have symptoms (84.2% vs 62.5%; p = 0.03) such as dyspnea (62.1% vs 37.5%; p = 0.04) and had a history of prior warfarin use (22.4% vs 0.0%; p = 0.01). Also, patients in the late PE group were more likely to have history of DVT although there was no statistically significant difference between the two groups (p = 0.05). The other clinical presentations, risk factors, and thrombophilic disorders did not differ significantly among the study groups.

Table 3 shows the radiological findings and management. Transthoracic echocardiography was performed in 45 (54.9%) patients. The most frequent abnormalities were right ventricular (RV) dilation in 11.0% of the cases, RV wall hypokinesis in six patients (7.3%), and dilation of pulmonary arteries in two patients (2.4%). Mobile thrombus in the right ventricle was seen in 1 patient (1.2%). It was diagnosed as infective endocarditis. The mean ejection fraction was 53+/-11 and 3 out of 6 cases with low ejection fraction died. Echocardiography was done in 10 (41%) cases with early PE and 35 (60.3%) with late PE.

**Table 3:** Radiological Findings and Management in Early and Late Pulmonary Embolism (PE)

Variables	Overall (n=82)	Early PE (≤4 Days) n=24	Late PE (>4 Days) n=58	P value
**Echocardiography findings:**	45 (54.9%)	10 (41.7%)	35 (60.3%)	0.12
Ejection fraction (%)	53.5±10.9	50.6±11.6	54.3±10.8	0.35
Right ventricular dilation	9 (11.0%)	1 (4.2%)	8 (13.8%)	0.20
RV wall hypokinesis	6 (7.3%)	1 (4.2%)	5 (8.6%)	0.48
Dilation of pulmonary arteries	2 (2.4%)	1 (4.2%)	1 (1.7%)	0.51
Mobile thrombi in right-heart chambers	1 (1.2%)	0 (0.0%)	1 (1.7%)	0.51
Inferior vena cava Filter	10 (12.2%)	2 (8.3%)	8 (13.8%)	0.49
**D-dimer test (n=76)**	33 (43.4%)	8 (34.8%)	25 (47.2%)	0.31
**Troponin test positive (n=53)**	19 (35.8%)	4 (23.5%)	15 (41.7%)	0.19
**CT angiography findings:**				
Main branch (unilateral thrombi)	18 (22.0%)	7 (29.2%)	11 (19.0%)	0.31
Main branch (Bilateral thrombi)	22 (26.8%)	4 (16.7%)	18 (31.0%)	0.18
Lobar & Segmental thrombi	53 (64.6%)	16 (66.7%)	37 (63.8%)	0.80
Sub-segmental thrombi	20 (24.4%)	10 (41.7%)	10 (17.2%)	0.01
Isolated segmental	21 (25.6%)	5 (20.8%)	16 (27.6)	0.52
Isolated lobar and Segmental thrombi	37 (45.1%)	9 (37.5%)	28 (48.3%)	0.37
Isolated sub-segmental thrombi	6 (7.3%)	3 (12.5%)	3 (5.2%)	0.24
**Wells Score**	3.9±1.8	3.3±1.8	4.2±1.8	0.08
**Revised Geneva score**	6.8±4.2	6.9±4.3	6.6±4.1	0.27
**Simplified PESI**	0.78±0.77	0.55±0.8	0.88±0.74	0.09
DVT on Ultrasonography (n=75)	14 (18.7%)	1 (4.8%)	13 (24.1%)	0.05
DVT on follow-up after PE	3 (3.7%)	0 (0.0%)	3 (5.2%)	0.25
**Prophylactic anticoagulation**	65 (79.3%)	20 (83.3%)	45 (77.6%)	0.55
**Time to Prophylaxis:**				
≤24 hours	28 (45.2%)	10 (47.6%)	18 (43.9%)	0.90 for all
24–48 hours	17 (27.4%)	6 (28.6%)	11 (26.8%)	
>48 hours	17 (27.4%)	5 (23.8%)	12 (29.3%)	
**Dalteparin prophylaxis **	52 (63.4%)	13 (54.2%)	39 (67.2%)	0.26
**Enoxaparin prophylaxis **	13 (15.9%)	6 (25.0%)	7 (12.1%)	0.14
**Anticoagulant’s treatment:**				
Enoxaparin	59 (72.0%)	17 (70.8%)	42 (72.4%)	0.88
Warfarin	16 (19.5%)	2 (8.3%)	14 (21.1%)	0.10
Heparin	14 (17.1%)	4 (16.7%)	10 (17.2%)	0.95
Dalteparin	4 (4.9%)	1 (4.2%)	3 (5.2%)	0.84
**Other management:**				
Thrombolytic therapy	8 (9.8%)	1 (4.2%)	7 (12.1%)	0.27
Thrombectomy	2 (2.4%)	0 (0.0%)	2 (3.4%)	0.35

The D-dimer was tested in 76 cases after the clinical suspicion of PE and was found positive in 33 (43.4%) cases. The cardiac troponin was positive in 35.8% cases.

**Figure 1:**
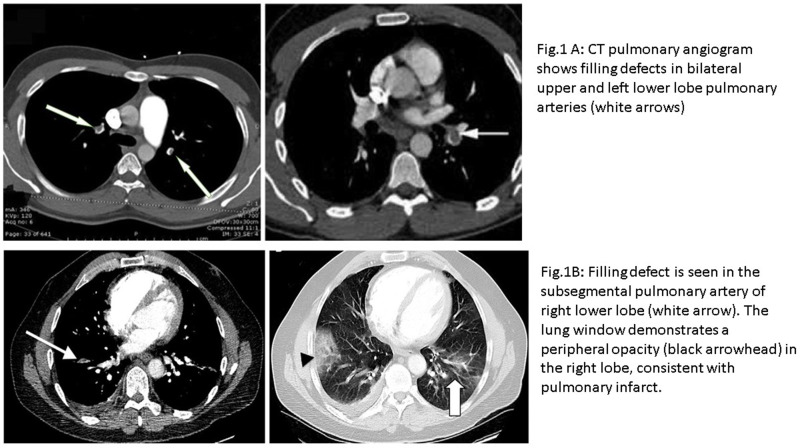
Examples of segmental and subsegmental pulmonary embolism by CTPA: (**A**) filling defects in bilateral upper and left lower lobe pulmonary arteries (white arrows). (**B**) Filling defect is seen in the subsegmental pulmonary artery of right lower lobe (white arrow). The lung window demonstrates a peripheral opacity (black arrowhead) in the right lobe, consistent with pulmonary infarct.

The CTPA was done in all patients to diagnose PE (Figure 1 shows examples of segmental and subsegmental PE). The findings revealed that 22 patients had main branch bilateral thrombi (26.8%), 18 (22.0%) had main branch unilateral thrombi and sub-segmental thrombi were identified in 24.4% cases. Six patients were found to have isolated sub-segmental PE. The two groups did not differ significantly with respect to the findings of echocardiography and CTPA except for the rate of sub-segmental thrombi which was significantly higher in the early PE group (41.7% vs 17.2%; p = 0.01). Estimates of the clinical probability of PE were performed according to the Wells′ score, Geneva revised score and Simplified PESI, which was found to be comparable among the two groups.

We did not perform DVT surveillance by Doppler ultrasonography before PE in our institution; however, most of patients underwent Doppler ultrasonography after the diagnosis of PE. Ultrasonography for DVT screening was done in 75 cases after the clinical suspicion of which 14 cases had DVT and 3 cases had DVT on follow-up after PE. Sixty-five (79.3%) patients received prophylactic anticoagulation (mainly dalteparin and enoxaparin). It was initiated within the first 24 h in 47.6 of the early PE patients and 43.9 of the late PE. Eight (9.8%) patients received thrombolytic therapy while 2 (2.4%) patients had thrombectomy in the operation theatre. The common initial treatment regimens were enoxaparin (72.0%), warfarin (19.5%), and unfractionated heparin (17.1%). The frequency of prophylactic anticoagulation, time to prophylaxis and anticoagulant therapy were comparable among the two groups.

Table 4 shows the comparison of complications and outcomes based on the time of PE.

**Table 4:** Complications and Outcomes in Early and Late Pulmonary Embolism

Variables	Overall (n=82)	Early PE (≤4 days) n=24	Late PE (>4 Days) n=58	P value
Heart rate (bpm)	104.8±21.6	103.9±22.7	105.1±21.2	0.82
Respiratory rate	23.2±7.6	19.7±5.7	25.0±7.9	0.01
Systolic blood pressure	121.0±21.9	122.8±20.4	120.3±22.6	0.67
Diastolic blood pressure	74.2±13.8	75.2±16.1	73.7±12.9	0.67
Oxygen saturation (%)	96.2±0.3	96.6±5.1	96.0±4.9	0.68
Shock Index	0.9±0.3	0.8±0.4	0.9±0.3	0.86
PaO2/ FiO2 ratio	2.3±1.0	2.5±1.1	2.2±0.9	0.44
**Intubation**	30 (36.6%)	7 (29.2%)	23 (39.7%)	0.37
**Tranexamic acid**	3 (3.7%)	0 (0.0%)	3 (5.2%)	0.25
**Units of Transfusion**	5 (1–19)	2.5 (1–17)	8 (1–19)	0.19
**Massive transfusion**	18 (22.5%)	4 (16.7%)	14 (25.0%)	0.41
**ICU length of stay**	12 (1–54)	5(1–42)	20 (1–54)	0.009
**Ventilatory days**	13 (1–84)	2 (1–42)	14 (2–84)	0.16
**Hospital length of stay**	27 (1–245)	16.5 (1–56)	32 (1–245)	0.03
**Complications:**				
Pneumonia	12 (14.6%)	2 (8.3%)	10 (17.2%)	0.29
Sepsis	7 (8.5%)	1 (4.2%)	6 (10.3%)	0.36
Recurrent DVT	5 (6.1%)	0 (0.0%)	5 (8.6%)	0.13
Multiorgan failure	1 (1.2%)	0 (0.0%)	1 (1.7%)	0.51
**In-hospital mortality**	4 (4.9%)	1 (4.2%)	3 (5.2%)	0.84
**Death after discharge**	1 (1.2%)	0 (0.0%)	1 (1.7%)	0.51
**Follow up Days**	90 (8–3145)	60 (10–1000)	180 (8–3145)	0.006

**Abbreviations**: PaO2, partial pressure of oxygen; FiO2, fraction of inspired oxygen.

The initial vital signs on admission were similar among the two groups except for respiratory rate which was significantly higher in the late PE group (25.0 ± 7.9 vs 19.7 ± 5.7; p = 0.01). Those with late PE had prolonged median ICU [20 (1–54) vs 5 (1–42); p = 0.009] and hospital [32 (1–245) vs 16.5 (1–56); p = 0.03] length of stay, compared to those with early PE. The early and late PE groups had similar rate of intubation, use of vasopressor/inotropes, tranexamic acid, need for blood transfusion, MT and in-hospital complications. During hospitalization, 4 (4.9%) patients died due to main pulmonary artery thrombosis; of which one (4.2%) died in the early group and 3 (5.2%) died in the late group (p = 0.84). Only one patient died after discharge from the hospital.

Table 5 shows the multivariable logistic regression analysis for early PE. After adjusting for the potential confounders, surgery within 24 hours of admission [age-gender-adjusted OR 37.58 (95% CI 3.393–416.20), p = 0.003] was found to be independent predictor of early PE in trauma patients.

**Table 5:** Multivariate Regression Analysis for Predictor of Early Pulmonary Embolism

Variables	Age-Gender-Adjusted Odds Ratio	95% Confidence Interval	P-value
Body mass index*	1.020	0.894–1.163	0.7711
**Surgery (<24 hours)**	37.58	3.393–416.20	0.003
**Long bone fracture**	0.704	0.138–3.604	0.674
Glasgow coma scale*	1.167	0.974–1.399	0.094
Injury severity score*	1.031	0.968–1.097	0.346
**Massive transfusion**	0.37	0.054–2.491	0.304

**Note**: *Continuous variable.

## Discussion

Although it is rare, venous thromboembolism (DVT and/or PE) is a potential sequela of injury that may result from the full dislodging of a clot in a deep vein of the limbs or from de novo thrombosis in the pulmonary artery as a result of post-traumatic inflammation.^22^,^23^ PE is a particularly serious problem in trauma patients, who are at high risk due to immobilization, surgical treatments, fear of bleeding, and direct tissue damage.^24^ Furthermore, current evidence suggests that PEs may arise within few days or may even occur immediately post-trauma.^25^ The outcome of PE can be improved considerably with early diagnosis and management. Our study sought to identify risk factors and clinico-radiological parameters associated with early onset PE following trauma. To our knowledge, only few investigators have reported potential risk factors of early PE after injury. The literature suggests that early PE is considered if occurs within 3 to 4 days after injury, this could be explained by how early the dual regulation of inflammation and thrombosis in the post-trauma period is.^24^,^26^ The occurrence of PE in trauma patients within 72 h represented 41.5% of the total VTE.^24^ Therefore, the present study considered early PE to occur within 4 days of hospitalization; based on this, 29% of our post-trauma PE patients developed early PE, and 71% had late PE. Earlier studies reported an incidence of early post-traumatic PE as being between 10% and 42%.^19^

Earlier, lower limb DVT was considered the main source of thrombus resulting in PE. Contemporary studies have identified that PE could occur without documented DVT and could be considered as a distinct entity.^14^,^27^,^28^ An earlier study by Velmahos et al^22^ demonstrated that most PE patients did not have evidence of DVT screened by CT venography of the pelvis and proximal lower extremity veins. PE could occur de novo within the lungs. The authors also found that some PE patients had pelvic or proximal DVT. Our study also supports this assertion, as the majority (75 out of 82) of our patients underwent screening by duplex ultrasonography after PE diagnosis. Only 18.7% were found positive for DVT and all had late PE. A study by van Langevelde et al^29^ used total body MRI to demonstrate that 55% of PE patients diagnosed by CT scan had no thrombus. They hypothesized that thrombi may originate from either a cardiac origin, complete dislodgement of a DVT or local inflammation of the lung vasculature.

The reported incidence of PE after trauma has increased in recent years, but the mortality has decreased significantly, likely due to improvement in radiological diagnosis and better management. PE incidence among trauma patients varies widely, ranging from 0.35% to 24%.^30^,^31^ An earlier study from our centre reported trauma-related DVT prevalence to be 8.5% and PE to be 10.9%.^32^ It has been shown that trauma patients are at increased risk of developing VTE.^33^ Those with PE are thought to have specific risk factors and clinical outcomes. This could be attributed to multiple factors such as age, comorbidities, type of injury, severity of injury, head and long bone fractures, immobility, blood transfusion and need for mechanical ventilation.^30^ In our study, patients in the early PE group were 2 years younger than those in the late group with no statistically significant difference. In contrast, a recent study reported that patients with early PE were older when compared to a late PE group.^34^ Our study found that early PE is frequent in a subset of the trauma population and has specific risk factors compared to late PE. O’Malley and Ross^35^ suggested that injured patients with respiratory compromise are at higher risk for developing early PE. Menaker et al^15^ showed that the incidence of early PE in trauma (within 4 days) was 37% and mostly occurred in the absence of long bone and spine fracture. In our study, a higher proportion of patients with long bone fractures (67%) developed PE overall, but there was no significant association with early PE. This contrasts with findings by Brakenridge et al^16^ who reported that fractures of long bones were found to be the sole independent risk factor for early PE. Whereas patients in the late PE groups had a higher ISS, sustained severe head or chest injuries, and experienced a delay in the commencement of pharmacological prophylaxis of more than 24 hours. long bone fractures were not associated with an increased risk of PE within the first 7 days of injury.^35^ The authors found pelvic fractures, age >55 years, severe single or multiple system trauma, and cannulation of central veins as risk factors of early PE. Knudson et al^1^ identified severe chest injury stimulating localized inflammation as a possible aetiology for PE. However, we found no significant difference in the frequency of chest injury among early and late PE groups in our study. Our assertion that some post traumatic PEs occur with a different underlying pathophysiology is nevertheless in line with Knudson’s conclusions. The present study showed that prior history of warfarin intake was associated with no incidence of early PE (0.0%) in comparison to 22.4% incidence of late PE. Prolonged ICU and hospital length of stay were associated with late PE. Moreover, the subsegmental thrombi was the predominate finding in the early PE (42% vs 17% in late PE) in our study. Although CTPA perfectly improves the detection of subsegmental PE (in which patients are often stable with favourable biomarker and echocardiographic findings), the mortality has been minimally changed.^36^

Our study found that surgery (within 24 hours) is an independent predictor of early PE on multivariate analysis, with an adjusted odds ratio of 37. An earlier study identified major operative procedures as a major independent risk factor for VTE, like our observation. However, the timing was not mentioned.^30^

A nested case-control study from Japan including 719 trauma patients with PE showed that 86% of patients with PE developed it post-surgery, the most common being bone fixation.^37^ Another study reported obesity, high SOFA score and surgical procedures as an independent predictor of early PE.^38^ Bahloul et al^24^ found that fracture of the lower extremities was commonly associated with early PE.

Prophylactic management of VTE for trauma patients remains the standard of care but may increase the risk of bleeding. Our institution has an aggressive regimen for VTE prophylaxis that is largely based on the use of low molecular weight heparin as soon as the patient is not actively bleed and has no coagulopathy (TBI patients not requiring blood products and cleared by a neurosurgeon). Anticoagulant treatment consists mainly of low molecular weight heparin with a pneumatic pressure pump on the lower limbs, if there are no fractures. In case of bleeding risk and no clearance by neurosurgeon in head trauma, an IVC filter is placed.

In our study, although time to chemical prophylaxis was comparable among the two groups, those with late PE were more likely to have delayed prophylaxis. An earlier study demonstrated that delay in chemical prophylaxis was observed in late PE which is consistent with our findings.^14^ The reasons for the delay were high-grade solid organ injuries, ongoing bleeding, head and spinal injuries and early discharge with the inability to ambulate.

Concomitant DVT was identified in 18.7% of cases in our study which agrees with an earlier study by Knudson et al^30^ which reported that 16% of PE cases were diagnosed to have DVT. However, establishing the link between DVT and PE needs further studies. Coleman et al^3^ showed that patients receiving a blood transfusion were at a greater risk of late PE. However, no significant difference was observed for the need of blood transfusion and MT among the two groups in our study. Gambhir et al^17^ reported that above knee DVT and blood transfusion were significant risk factors for late PE while smoking history was significant in early PE. According to published reports, fatality rates from trauma-related PE range from 17% to 26%.^15^ In our study, of the 82 patients diagnosed with PE, 4 (4.9%) died during hospitalization, and one (1.2%) death occurred after discharge. Lastly, in comparison with trauma incidents, the most recent study on PE in Qatar among non-trauma population showed a crude incidence rate of 88 cases per 100000 per year and one-third of them occurred in-hospital, however the majority was reported in the surgical wards.^39^

## Limitations

The present study has few limitations, due to its retrospective design. The small sample size and single-institution study could affect the generalizability of the findings and power of the study. Moreover, due to the observational nature, causal correlations between risk factors for PE and outcome could not be established. Because of small number of outcome (early PE), we included only 8 variables in the multivariable analysis such as the body mass index, early surgery, long bone fractures, brain injury (using GCS), polytrauma (using ISS) and the use of massive blood transfusion in addition to age and gender, as we do believe that these are the most important relevant factors particularly that we noticed that the coagulation factors and D-dimer were not statistically differed between the two groups. However, the results should be cautiously considered. Moreover, our data did not captured information on the occurrence of fat embolism (especially among early PE patients and post-surgery) which is common after long bone and pelvic fracture with an incidence rate between <1% and 29%.^40^ Our institution has initiated thromboelastographic (ROTEM) for the initial assessment of trauma patients, which will help us understand the association between PE and coagulopathy. Finally, there is a chance that the disease burden is underrepresented, as some individuals may have been diagnosed with PE after being discharged from the trauma centre. Lack of routine post-mortem examination may underestimate the incidence of PE in our centre. However, data were retrieved from the only level 1 trauma centre in the country (Qatar has a total of 2.7 million inhabitants) that admits and treats 95% of all moderate-to-severe traumatic injuries. Of note, data from this centre are well validated internally and externally as it is a part of the National Trauma Data Bank (NTDB) and compliant with the standards of the American College of Surgeons Trauma Quality Improvement Program (ACS-TQIP) in the USA.^41^

## Conclusions

One-third of the PE cases occurred early post-trauma and surgery within the first 24 hours of admission was independently associated with early PE. A prospective study is needed to develop an objective risk assessment system for early and late PE. A high index of suspicion for early PE is warranted in trauma patients who undergo early surgical intervention. The precise mechanism of PE formation in trauma seems to be independent of the occurrence of DVT and deserves further in vitro and in vivo study to reduce the risk of this feared complication. Lastly, delay in chemoprophylaxis should be minimized, as these could lead to higher rates of PE in trauma patients.

## Data Sharing Statement

All data were presented in the manuscript and tables.
